# Osteoarticular Sporotrichosis in a Brazilian Endemic Setting: Associated Factors and Outcomes

**DOI:** 10.1111/myc.70211

**Published:** 2026-07-24

**Authors:** Vanessa Caroline Randi Magalhães, Alexandre Sampaio Moura, Fábio do Nascimento Sales, Salene Angelini Colombo, Renata Eliane de Ávila, Jorge Miguel Schettino César, Diana Manzi Viegas, Amanda Cristina Silva Tardelli Pizarro, Ana Cláudia Lyon, Silvia Hees Carvalho, Maria Rita Teixeira Dutra, Virginia Antunes Andrade, Maria Isabel Azevedo, Nalu Teixeira Aguiar Peres, Daniel Assis Santos

**Affiliations:** ^1^ Department of Microbiology, Institute of Biological Sciences Universidade Federal de Minas Gerais Belo Horizonte Brazil; ^2^ Hospital Eduardo de Menezes Fundação Hospitalar do Estado de Minas Gerais (FHEMIG) Belo Horizonte Brazil; ^3^ Department of Preventive Veterinary Medicine, School of Veterinary Universidade Federal de Minas Gerais Belo Horizonte Brazil; ^4^ Hospital Júlia Kubitschek Fundação Hospitalar do Estado de Minas Gerais (FHEMIG) Belo Horizonte Brazil; ^5^ Hospital João XXIII Fundação Hospitalar do Estado de Minas Gerais (FHEMIG) Belo Horizonte Brazil; ^6^ Brazilian National Institute of Science and Technology in Human Pathogenic Fungi (INCT‐FUNVIR) São Paulo Brazil

**Keywords:** Brazilian cohort, diabetes, risk factors, *Sporothrix brasiliensis*, sporotrichosis osteoarticular

## Abstract

**Background:**

Osteoarticular infection is an emerging complication of sporotrichosis, often leading to a significant increase in morbidity and longer treatment.

**Methods:**

We conducted a retrospective cohort study of osteoarticular sporotrichosis (OS) in southeastern Brazil (2014–2024) using clinical, epidemiological, and molecular data.

**Results:**

Among 252 patients, 20 (7.9%) had OS, including 10 (4%) with osteomyelitis. The median age of patients with OS was 58 years (IQR 39–64; range 18–83) and 55% were male. Hypertension (45%), diabetes (40%), alcohol use (40%), smoking (40%), and HIV infection (15%) were the most frequent conditions identified. Cutaneous lesions occurred in 95% of cases, and 90% of patients presented with arthralgia and edema. Multifocal OS was observed in 50% of cases, most commonly affecting the knees, fingers, and wrists. The mean time to diagnosis was 14.2 months. Most patients were treated with itraconazole combined with amphotericin B; 13 required surgical intervention, including 2 amputations. The mean treatment duration was 21 months. Multisystem involvement due to sporotrichosis was observed in 45% of cases, including pulmonary involvement. Mortality was 15%. Diabetes (OR = 4.11; 95% CI, 1.50–11.38; *p* = 0.006) was independently associated with OS.

**Conclusions:**

OS is associated with substantial morbidity and prolonged treatment. Diabetes may increase the risk of OS, while delayed diagnosis jeopardizes outcomes, underscoring the need for early management.

## 1 Introduction

Sporotrichosis is an implantation mycosis caused by fungi of the genus *Sporothrix*. Although it has a worldwide distribution, it is particularly prevalent in Brazil [1, 2, 3]. The disease primarily manifests as cutaneous lesions [4]. However, there has been a significant increase in the incidence of extracutaneous forms, primarily associated with disseminated disease [3, 5, 6]. The emergence of severe and atypical manifestations of sporotrichosis has been attributed to host immunosuppression, which is commonly associated with HIV infection [7, 8]. These cases have predominantly been associated with *Sporothrix brasiliensis*, widely recognized as the most virulent species within the genus [9].

Fungal infections are uncommon causes of bone disease [10]. However, osteoarticular sporotrichosis (OS) is becoming increasingly prevalent, particularly in hyperendemic areas, typically presenting as osteomyelitis or arthritis [11, 12]. OS, including its severe forms, may be underdiagnosed or late diagnosed, mainly due to non‐specific clinical manifestations [12, 13], especially when there is no apparent history of cutaneous trauma [14, 15, 16]. In addition, current treatment guidelines [17, 18] do not differentiate between unifocal (lesions in a single anatomical site) and multifocal (lesions in more than one anatomical site) osteoarticular infections caused by *Sporothrix* spp. [12], jeopardizing prognostic and therapeutic management.

Improving understanding of the clinical course of OS and its associated factors may enable earlier diagnosis and more effective therapeutic interventions, thereby reducing the disease burden in vulnerable populations. Therefore, this study aimed to evaluate the clinical and epidemiological characteristics, diagnostic approaches, and therapeutic strategies for OS in patients attended in Southeastern Brazil.

## 2 Methods

This retrospective cohort study analysed epidemiological and clinical data from a Reference Center for Infectious Diseases (Hospital Eduardo de Menezes, Fundação Hospitalar do Estado de Minas Gerais—HEM–FHEMIG) in Brazil, from 2014 to 2024. The study was approved by the Ethics Committee (CAAE 00883118.0.0000.5149 and CAAE 00883118.0.3001.5124). Data were obtained from patients' medical records including information on demography, clinical classification of sporotrichosis, exposure history, comorbidities, clinical manifestations, imaging findings, microbiological and histopathological data, treatments, and outcomes. Patients were followed for an additional 6 months after completion of the cohort study. Statistical analyses were performed using Epi Info 3.5.3 (Centers for Disease Control and Prevention).

Among 252 patients with sporotrichosis, 52 with suspected osteoarticular involvement, defined by symptoms such as joint pain or swelling, were selected for the study. OS was defined by imaging evidence of osteomyelitis, arthritis, tenosynovitis, synovitis, or bursitis, and by isolation of the fungus from osteoarticular structures [12]. Confirmed cases of OS required the isolation of *Sporothrix* spp. from bone or synovial fluid. In contrast, probable cases of OS were defined as those with microbiological or histopathological confirmation from adjacent or systemic samples combined with compatible clinical and radiological findings [10]. Articular reaction was defined as a mild inflammatory process without radiological changes and without isolation of *Sporothrix* spp. from osteoarticular samples [18]. Patients with incomplete OS diagnostic evaluation or who discontinued antifungal therapy were excluded.

Descriptive statistics were used to summarize data from OS cases. Categorical variables were reported as absolute and relative frequencies, while quantitative variables were represented as means and standard deviations.

Bivariate and multivariate unconditional logistic regression analyses were performed to assess factors associated with OS in a cohort of 252 patients, comparing OS cases (confirmed and probable) versus the remainder of the cohort (controls). Variables associated with OS with *p* < 0.20 in the univariate analysis were included in a multivariable model built by backward elimination, with statistical significance defined as *p* < 0.05.

Fungal isolates were cultured on Sabouraud Dextrose Agar at 25°C; dimorphism was assessed on Brain Heart Infusion agar at 37°C and 5% CO_2_. Identification was based on macroscopic and microscopic morphological features and confirmed by PCR [19].

## 3 Results

### 3.1 Epidemiological and Clinical Findings

**FIGURE 1:**
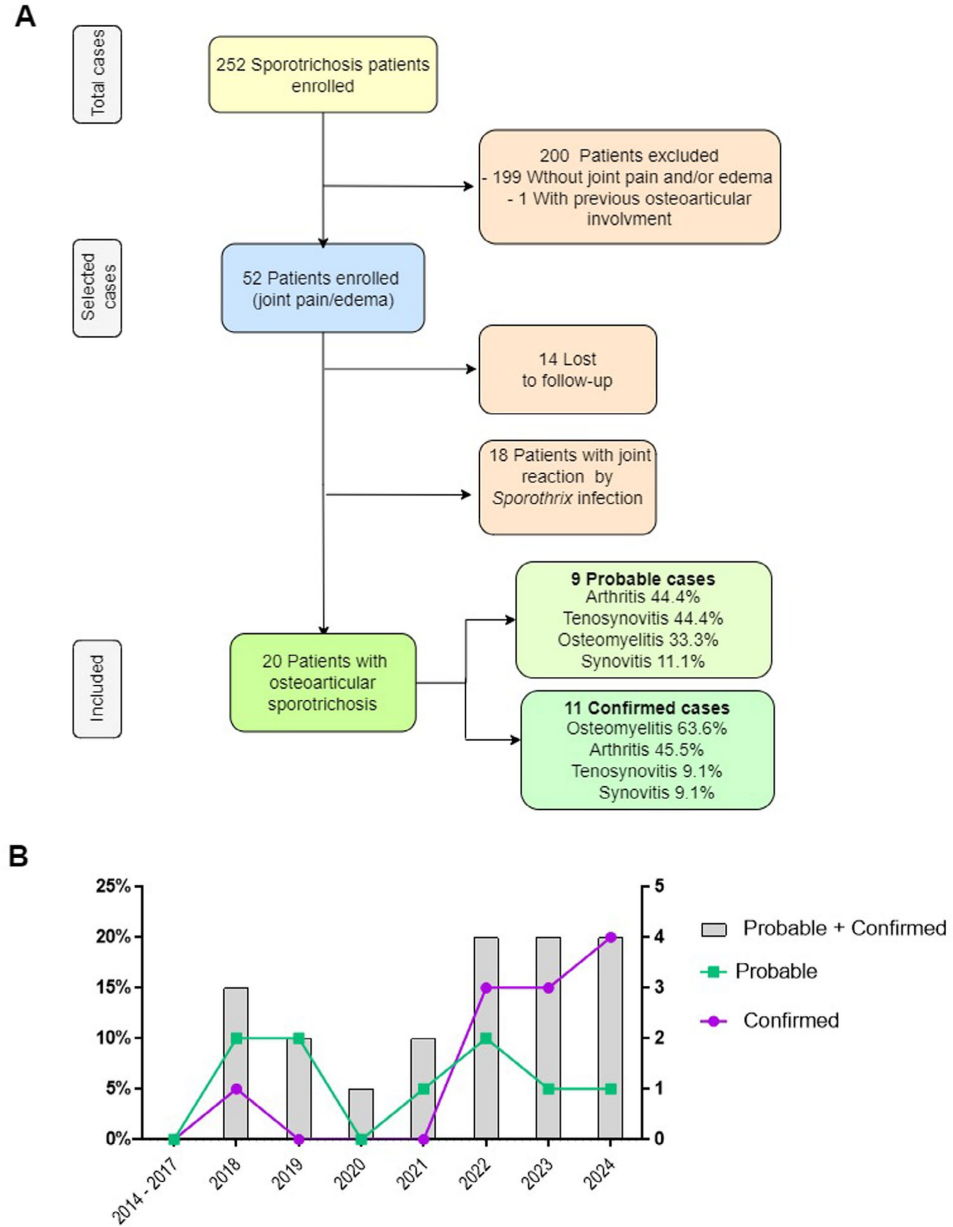
(A) Flowchart of patient inclusion criteria for this study. (B) Cases of osteoarticular sporotrichosis (OS) over 10 years (*n* = 20) defined as probable or confirmed.

In this study, 252 patients were diagnosed with sporotrichosis between 2014 and 2024, with a range of clinical manifestations. Of those cases, 20 (7.9%) had OS, including 10 cases (4%) of osteomyelitis. Figure 1A illustrates patient enrollment and the inclusion/exclusion criteria, and Figure 1B the temporal distribution of OS cases, classified as probable or confirmed. The first cases were reported in 2018, followed by a progressive increase in subsequent years.

The median age of patients with OS was 58 years (IQR 39–64; range 18–83), with a slight predominance of male patients (55%). Most patients (75%) reported contact with cats. Of those, 66.7% had contact with a sick cat, and 40% recalled a traumatic contact with felines. Bites were reported in 26.7% of cases, and scratches in 13.3%. Furthermore, two patients (10%) reported contact with dogs, two (10%) were employed as construction workers, and two (10%) reported engaging in gardening activities. Table 1 provides an overview of the epidemiological and clinical characteristics of the OS cases.

**TABLE 1:** Epidemiological and clinical features of osteoarticular sporotrichosis cases.

	All *n* = 20 (%)
Median age (IRQ)	58 (39–64)
Male	11/20 (55)
Contact with cats	15/20 (75)
Bites and/or scratches	6/15 (40)
Non‐traumatic^a^	8/15 (53)
Contact with dogs	2/20 (10)
Construction work	2/20 (10)
Gardening activities	2/20 (10)
Hypertension	9/20 (45)
Diabetes	8/20 (40)
Alcohol use	8/20 (40)
Smoking	7/20 (35)
HIV	3/20 (15)
Cutaneous lesions	19/20 (95)
Nodular lesion	12/19 (63.1)
Wound pain	10/19 (52.6)
Secondary bacterial infection	7/19 (36.8)
Arthralgia	18/20 (90)
Multiple joints	15/18 (83.3)
Knees	8/18 (44.4)
Fingers	6/18 (33.3)
Wrists	6/18 (33.3)
Arms	3/18 (16.7)
Legs	3/18 (16.7)
Ankles	3/18 (16.7)
Feet	2/18 (11.1)
Costal arches	2/18 (11.1)
Elbows	1/18 (5.6)
Shoulders	1/18 (5.6)
Pulmonary involvement	6/20 (30)
Pulmonary and neurological involvement	1/20 (5)
Pulmonary, neurological and ocular involvement	1/20 (5)
Ocular involvement	1/20 (5)
Fever	9/20 (45)
Weight loss	7/20 (35)
Multifocal osteoarticular involvement	10/20 (50)

^a^ Cohabitation with cats (± direct handling).

Nine patients (45%) had hypertension, eight (40%) had diabetes, eight (40%) reported alcohol use, seven (35%) were smokers, two (10%) had undergone bariatric surgery, and three (15%) were people living with HIV (PLWH). Among the PLWH, CD4+ T cell counts at the time of sporotrichosis diagnosis ranged from 5 to 204 cells/mm^3^, with a mean of 85 ± 105 cells/mm^3^. Viral loads ranged from 30,793 to 58,307 copies/mL.

Most patients (90%) presented with arthralgia, and the pain affected multiple joints, being associated with edema in 50% (9/18) of cases. The most affected sites were the knees (44.4%), followed by the fingers and wrists (33.3% each), arms, legs, and ankles (16.7% each), feet and costal arches (11.1% each), elbows and shoulders (5.6% each). In addition to osteoarticular involvement, six patients (30%) presented with pulmonary involvement, one (5%) with both pulmonary and neurological involvement, one (5%) with pulmonary, neurological, and ocular involvement, and another (5%) with ocular involvement. Overall, multisystem involvement was observed in 45% of cases. The most common non‐specific signs and symptoms were fever (45%) and weight loss (35%). Table 2 presents the individual clinical characteristics, transmission routes, initial clinical presentation, time to OS diagnosis, treatment, and outcomes for the 20 OS patients.

**TABLE 2:** Clinical characteristics, transmission routes, initial clinical form, diagnosis time, treatment, and outcomes of patients with osteoarticular sporotrichosis.

Case/Classification of OS	Sex/Age	Predisposing factor	Probable route of infection	Initial clinical form	Affected location	Osteoarticular involvement	Systemic involvement	Time to diagnosis of OS (in months)^a^	Antifungal treatment	Surgical	Outcome
1/Probable	F/63	Diabetes	Contact with cats and local trauma	LS followed by widespread nodular lesions and joint pain	R wrist (tenosynovitis)	Unifocal	No	10	ITZ 200 mg/day, 6 months; AMB (l) ~1.5 g, AMB (d) ~0.4 g	No	Cure
2/Probable	F/68	Diabetes, hypertension, alcohol ingestion and smoking	Contact with cats	LS	Widespread nodular lesions and joint pain. Costal arch (osteomyelitis)	Unifocal	Pulmonary	72	No	No	Death
3/Probable	F/41	Alcohol ingestion and smoking	Unknown	Widespread nodular lesions and joint pain	L knee (arthritis)	Unifocal	Pulmonary	< 1	ITZ 600 mg/day, 8 days; ITZ 200 mg/day, 10 months; ITZ 400 mg/day, 2 months; AMB (d) ~0.85 g	No	Cure
4/Probable	F/87	Hypertension	Cat bite	LS	R radio, R 2nd and 3rd fingers (osteomyelitis), R wrist (tenosynovitis)	Multifocal	Pulmonary	13	ITZ 200 mg/day, 21 months, ITZ 400 mg/day, 40 months; AMB (cl) ~2.8 g; AMB (d) ~0.7 g and AMB (l) ~4.5 g	Yes	Cure
5/Probable	M/25	Alcohol ingestion	Cat bite	CS	R 1st and 2nd fingers (tenosynovitis)	Multifocal	No	2	ITZ 100 mg/day, 1 months; ITZ 200 mg/day, 1 months; ITZ 400 mg/day, 63 months and AMB (d) ~0.4 g	Yes	Cure
6/Probable	M/46	Alcohol ingestion, smoking and HIV	Unknown	Widespread nodular lesions and joint pain	L 2nd finger (osteomyelitis), R 4th metatarsal (arthritis)	Multifocal	Pulmonary, neurological e ocular	6	ITZ 400 mg/day, 33 months; AMB (d) ~0.25 g; AMB (cl) ~2.8 g; AMB (l) ~1.4 g and AMB (l) ~0.28 g/day every 15 days, continuous use	Yes	On treatment
7/Probable	F/70	Hypertension and smoking	Unknown	Widespread nodular lesions and joint pain	Hands, hip and R shoulder (arthritis), R wrist (synovitis)	Multifocal	Pulmonary	4	ITZ 200 mg/day, 1 months and ITZ 400 mg/day, 11 months	No	Cure
8/Probable	F/35	Bariatric surgery	Cat scratch	LS followed by widespread nodular lesions and joint pain	L hand and L wrist (tenosynovitis)	Multifocal	No	2	ITZ 200 mg/day, 16 days; ITZ 400 mg/day, 4 months, and AMB (d) ~0.25 g	Yes	Cure
9/Probable	F/66	No	Cat bite	LS	R hand (arthritis)	Unifocal	No	2	ITZ 400 mg/day, 3 months	No	Cure
10/Confirmed	M/62	Diabetes, hypertension, alcohol ingestion and smoking	Contact with cats and soil	Widespread nodular lesions and joint pain	L 1st finger, L tibia (osteomyelitis) and L knee (arthritis)	Multifocal	No	< 1	ITZ 400 mg/day, 22 months; AMB (cl) ~4.6 g	Yes (Amputation)	Cure
11/Confirmed	M/24	HIV	Contact with cats	LS	L knee, L ankle and L foot (arthritis)	Multifocal	Pulmonary	22	ITZ 400 mg/day, 21 months (consolidation treatment). AMB (cl) ~1.0 g	No	Death
12/Confirmed	M/50	Hypertension, alcohol ingestion and smoking	Contact with cats and soil	Widespread nodular lesions and joint pain	R tibia (osteomyelitis)	Unifocal	Pulmonary	17	ITZ 400 mg/day, 16 months; AMB (d) ~0.8 g, AMB (l) ~1.8 g. ITZ 400 mg/day, 20 months; AMB (cl) ~8.4 g	Yes	On treatment
13/Confirmed	M/52	Diabetes and hypertension	Contact with cats and soil	Widespread nodular lesions and joint pain	L hand, L wrist (tenosynovitis), L 3rd and 4th fingers (synovitis)	Multifocal	No	5	ITZ 200 mg/day, 2 months; ITZ 400 mg/day, 11 months; AMB (cl) ~4.5 g	Yes	Cure
14/Confirmed	M/57	Diabetes, alcohol ingestion, smoking, bariatric surgery	Cat bite	Osteomyelitis	R 2nd metatarsal and 2nd toe (osteomyelitis)	Unifocal	No	84	ITZ 400 mg/day, 7 months	Yes (Amputation)	Cure
15/Confirmed	M/64	Diabetes and hypertension	Contact with cats	Widespread nodular lesions and joint pain	R elbow (arthritis)	Unifocal	Pulmonary	3	No	Yes	Death
16/Confirmed	M/60	Alcohol ingestion	Contact with sand Environment with cat (sand)	Widespread nodular lesions and joint pain	R ankle (osteomyelitis), L knee (arthritis) (August, 2022). L tibia and L femur (osteomyelitis) (May, 2023)	Multifocal	No	9	(1) ITZ 400 mg/day, 10 months. AMB (l) ~1.5 g. (2) ITZ 400 mg/day, 23 months (ongoing)	Yes	On treatment
17/Confirmed	M/36	HIV	Unknown	Pulmonary sporotrichosis	R knee (arthritis)	Unifocal	Pulmonary and neurological	3	ITZ 400 mg/day, 8 months (ongoing) AMB (cl) ~3.9 g	No	On treatment
18/Confirmed	M/64	Diabetes and hypertension	Unknown	Widespread nodular lesions and joint pain	R knee (osteomyelitis)	Unifocal	No	< 1	ITZ 200 mg/day, 1 months; ITZ 400 mg/day, 11 months; AMB (cl) ~3.2 g	Yes	On treatment
19/Confirmed	F/65	Diabetes and hypertension	Cat scratch	LS and joint pain	L knee (osteomyelitis) (January, 2024). R radius, R humerus and R ulna (osteomyelitis) (February, 2025)	Multifocal	No	20	ITZ 400 mg/day, 12 months AMB (cl) (ongoing)	Yes	On treatment
20/Confirmed	M/21	No	Contact with cats	LS followed by a single nodular lesion that is difficult to heal	7th costal arch (osteomyelitis)	Unifocal	No	6	ITZ 100 mg/day, 18 days, ITZ 400 mg/day, 10 months (ongoing) AMB (cl) 1.5 g	Yes	On treatment

Abbreviations: AMB, amphotericin B; cl, lipid complex formulation; CS cutaneous sporotrichosis; de, deoxycholate formulation; F, female; ITZ, itraconazole; L, left; l, liposomal formulation; LS, lymphocutaneous sporotrichosis; M, male; OS, osteoarticular sporotrichosis; R, right.

^a^ Time between the initial diagnosis of sporotrichosis and the OS diagnosis.

The interval between the initial diagnosis of sporotrichosis and the OS diagnosis ranged from less than 1 month to 7 years, with an average of 14.2 ± 22.8 months (Table 2). Notably, the shortest diagnostic intervals for OS were observed among patients unable to identify the source or onset of the infection. These individuals often presented with atypical symptoms, including disseminated nodular lesions and/or pulmonary involvement and advanced osteoarticular disease. Although half of the patients had multifocal OS, this proportion may be underestimated due to incomplete imaging and microbiological evaluation of all symptomatic sites.

### 3.2 Imaging Findings

The imaging protocols included computed tomography (8/18, 44.4%), magnetic resonance imaging (7/18, 38.9%), and both radiography and ultrasonography (6/18, 33.3% each). Imaging findings revealed osteomyelitis (9/18, 50%), septic arthritis (8/18, 44.4%), septic tenosynovitis (5/18, 27.8%), and synovitis (2/18, 11.1%). Soft tissue abscesses were the most common finding, occurring in 12/18 (66.7%) patients, followed by bone erosions (9/18, 50%), joint effusion (8/18, 44.4%), joint space narrowing (6/18, 33.3%), bone marrow edema (5/18, 27.8%), and periosteal reaction (1/18, 5.6%). Osteomyelitis most frequently involved the tibia and fingers (18.7% each), followed by the radius and knees (12.5% each), whereas septic arthritis predominantly affected the knees (46.2%).

### 3.3 Clinical, Microbiological, Histological, and Cytological Findings

**FIGURE 2:**
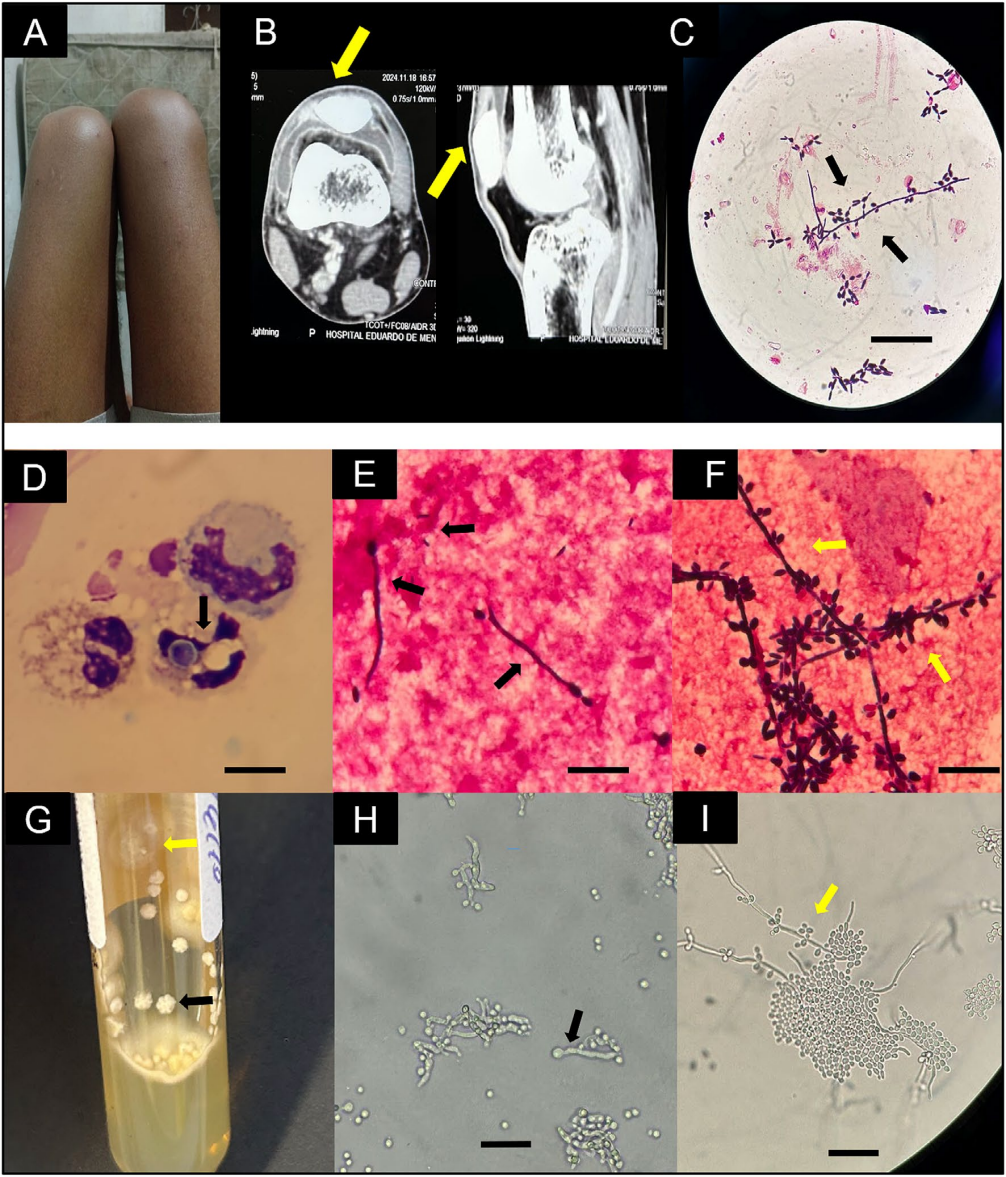
Representative pictures of osteoarticular sporotrichosis (OS) cases with confirmed microbiological tests. *Case 17* (A–C): PLWH being treated for pulmonary sporotrichosis without skin lesions. (A) Right knee edema. (B) CT scan of the right knee showing joint effusion (yellow arrow). (C) Hyphae with conidia of *S. brasiliensis* (arrows), stained with Panoptic, obtained from a synovial fluid culture after 4 days of incubation in liquid medium (BD BACTEC) at 35°C (bar: 15 μm). *Case 11* (D–I): PLWH on consolidation therapy for sporotrichosis for 22 months, presenting with recalcitrant OS. (D) *S. brasiliensis* yeast visualized inside a neutrophil in synovial fluid cytology, stained with Panoptic. (E, F) Yeast‐like cells, pseudohyphae (black arrows), and hyphae with conidia (yellow arrows) stained Gram‐negative after 4 days of incubation of synovial fluid in aerobic liquid medium (BD BACTEC) at 37°C (bar: 15 μm). (G) Two macroscopically distinct colonies of *S. brasiliensis* isolated from synovial fluid cultured on Sabouraud dextrose agar at 25°C for 10 days. (H–I) Microscopic analysis of the colonies; structures suggestive of pseudohyphae (black arrow) and septate hyphae with embedded conidia (yellow arrow) (bar: 15 μm).

Bone and synovial fluid cultures were essential for the OS diagnosis in patients without skin lesions, a circumstance in which sporotrichosis was not initially considered. One example was an HIV‐positive patient who developed left knee arthritis caused by *Sporothrix* infection, confirmed by synovial fluid culture, without any skin lesions or prior trauma (Figure 2A–C).

**FIGURE 3:**
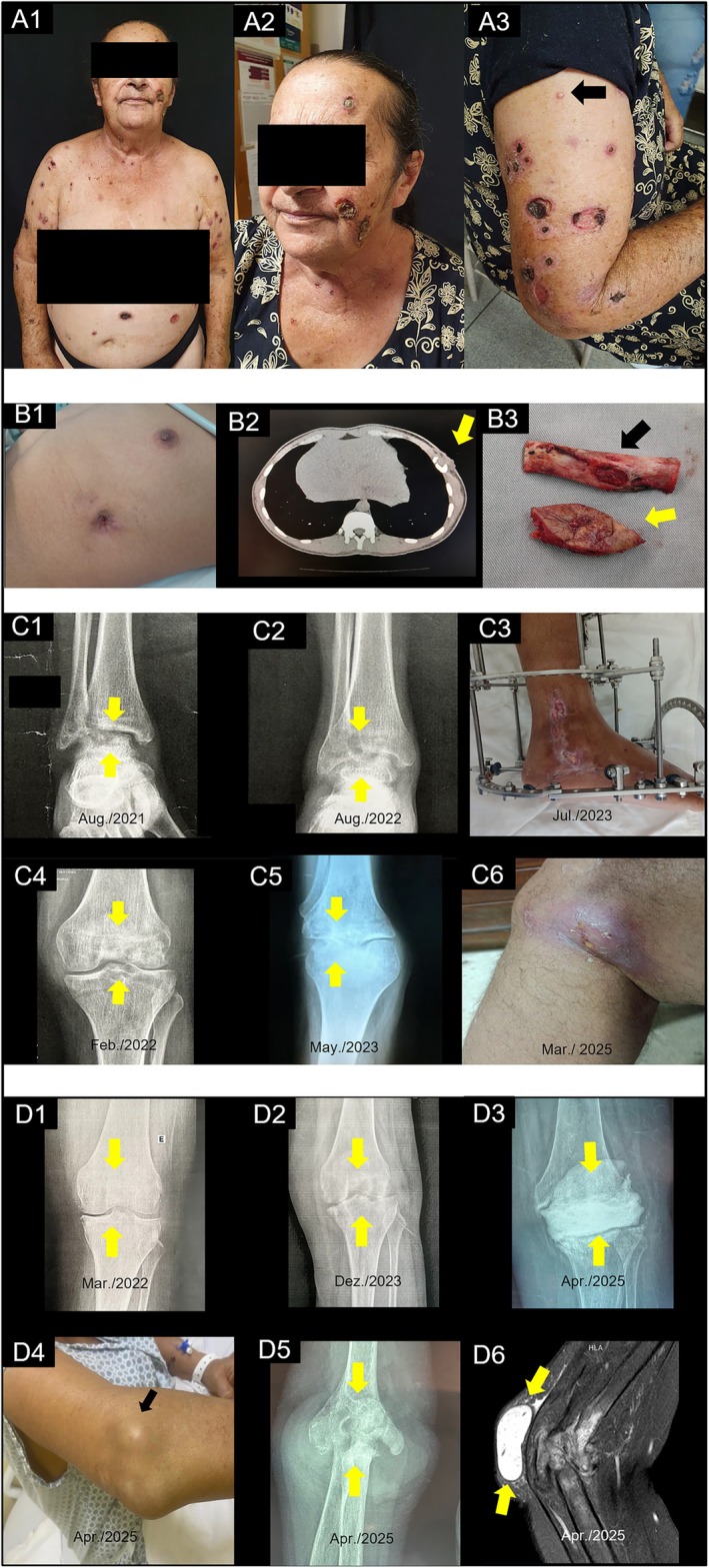
Representative pictures of OS patients. *Case 7*: Widespread nodular lesions throughout the body. (A1–A3) Lesions at different stages of progression and a new lesion (black arrow). *Case 20*: Osteomyelitis in the left 7th costal arch. (B1) Single slow‐healing sporotrichosis lesion. (B2) Chest computed tomography (CT) showing an osteolytic, permeative lesion with a periosteal reaction (yellow arrow). (B3) Bone fragment with osteolytic lesion (black arrow) and skin fragment (yellow arrow). *Case 16*: Multifocal OS. (C1) Right ankle arthritis with early joint space narrowing, bone marrow edema, and abscesses. (C2) One‐year follow‐up showing progression to osteomyelitis with joint space narrowing and extensive bone erosions. (C3) Ankle arthrodesis. (C4) Left knee arthritis with joint space narrowing and medial femoral and tibial erosions. (C5) Fifteen‐month follow‐up showing marked joint space narrowing and multiple large bone erosions. (C6) Draining skin lesions on the left knee. *Case 19*: Multifocal OS. (D1) Progressive left knee arthritis with joint space narrowing. (D2) Left knee osteomyelitis after 21 months, with narrowing and multiple erosions. (D3) Post‐surgery and orthopaedic cement in the left knee. (D4) Nodular lesion on the right elbow (black arrow). (D5) Progression to osteomyelitis of the left elbow after 57 months. (D6) Magnetic Resonance (MR) imaging showing a large amount of fluid located in the posterior region of the right elbow (yellow arrow).

Synovial fluid cytology typically showed a yellow, cloudy fluid without clot formation. Nucleated cell counts ranged from 960 to 3920 cells/μL, and red blood cell counts ranged from 240 to 6750 cells/μL. Neutrophils comprised 13%–82% of the cells, while lymphocytes comprised 18%–79%. Yeast‐like cells were observed intracellularly in Panoptic‐stained smears (Figure 2D). Furthermore, typical yeast cells (cigar‐shaped), pseudohyphae (Figure 2E), and filamentous forms with conidia (Figure 2F) were simultaneously observed in synovial fluid cultured in aerobic liquid medium at 37°C. In Case 11, two morphologically distinct colonies obtained from synovial fluid culture on Sabouraud agar at 25°C, both identified as *S. brasiliensis* , exhibited microscopic differences (Figure 2G–I).

*Sporothrix* spp. was isolated from at least one site in all patients, including cutaneous specimens, such as lesion exudate or skin biopsy (20/33, 60.6%), synovial fluid (6/33, 18.2%), bone biopsy (5/33, 15.1%), and deep tissue, blood, or sputum (1/33, 3% each). Twenty fungal isolates from 13 patients were identified as *S. brasiliensis* by PCR. Bacterial–fungal coinfection of bone tissue occurred in three patients, involving *Staphylococcus aureus* , *Escherichia coli* , *Streptococcus pyogenes* , and *Pseudomonas stutzeri* .

Histopathology of bone tissue from four patients revealed fragmented trabeculae in three (75%) cases and chronic granulomatous inflammation with caseous necrosis in one (25%) case.

Cutaneous lesions were observed in 95% of patients. In 63.1% (12/19), nodular lesions measuring 0.5–1.5 cm in diameter were diffusely distributed across the face, trunk, arms, and legs, with no clear association with any specific inoculation site (Figure 3A1–A3). Those disseminated lesions often developed after resolution of primary sporotrichosis, or in patients with no apparent history of cutaneous trauma. They frequently evolved into ulcers with purulent exudate. One patient, after healing of the disseminated lesions, presented a single slow‐healing lesion associated with osteomyelitis in the left seventh costal arch (Figure 3B1–B3).

Two patients presented with recalcitrant multifocal OS (Figure 3C1–C6 and D1–D6). The first patient (Case 16) involved a construction worker who denied direct contact with cats (contact with sand in an environment with cats) and presented with right ankle arthritis and disseminated nodular lesions, which progressed to osteomyelitis despite treatment with itraconazole (400 mg/day). Two years later, a draining fistula with purulent discharge and osteomyelitis of the left knee was observed, and treatment was resumed. The second patient (Case 19) represents a patient who sustained a cat scratch on the right thumb, resulting in a cutaneous lesion that was treated with debridement and itraconazole for 8 months. This patient later developed arthritis in the left knee and right elbow, which progressed to osteomyelitis in both sites.

### 3.4 Treatment and Outcomes

At the time of OS diagnosis, most patients (90%) received antifungal treatment. Two patients (Cases 2 and 15) were diagnosed late and died without receiving treatment. Treatment was prescribed based on the severity, as detailed in Table 2. Among the treated patients, itraconazole was used at doses ranging from 100 to 600 mg/day. The most common dose was 400 mg/day (59.2%), followed by 200 mg/day (29.6%). The predominant regimen was the combination of itraconazole and amphotericin B (83.3%). Thirteen patients (65%) underwent surgical treatment. Finger and toes amputation was required in two cases. Antibacterial therapy was administered in 65% of cases for the management of secondary bacterial skin infections in 76.9% of patients and osteoarticular coinfections in 23.1%. Corticosteroid therapy before the diagnosis was reported in four patients.

By the end of the study, 50% of patients were cured, with treatment durations ranging from 3 to 65 months (mean ± SD: 20.6 ± 23 months). Seven patients (35%) remained under treatment, with treatment durations ranging from 8 to 36 months (mean ± SD: 21.2 ± 10.6 months), considering that patients were followed for an additional 6 months after the end of the study period. Three (15%) died, two of whom had a delayed diagnosis.

The clinical manifestations in cured patients were tenosynovitis (5/10, 50%), arthritis (4/10, 40%), osteomyelitis (3/10, 30%), and synovitis (2/10, 20%), with the latter predominantly affecting the fingers and toes. Sixty percent of these cases required surgical intervention, including amputation in two patients.

On the other hand, among patients still undergoing treatment, osteomyelitis was the predominant manifestation (6/7, 85.7%), primarily affecting the knees and long bones, and surgical intervention was required in 85.7% of cases, with many undergoing multiple procedures at the same or different anatomical sites.

Osteoarticular sequelae were observed in 50% of patients and included loss of mobility and deformities (5/10, 50%), atypical gait (3/10, 30%), and paresthesia (3/10, 30%).

### 3.5 Predisposing Factors for OS

A univariate model identified the predisposing factors associated with OS. In the final model, diabetes (OR = 4.11; 95% CI = 1.50–11.38; *p* = 0.006) was independently associated with OS (Table 3).

**TABLE 3:** Association analysis of predisposing factors comparing confirmed and probable cases of osteoarticular sporotrichosis with the remainder of the cohort (controls).

Variable	Univariable model			Multivariable model**		
	OR	95% CI	*p*	OR	95% CI	*p*
Drinking alcohol	**2.85**	**(1.10–7.39)**	**0.031**			
Diabetes	**5.06**	**(1.90–13.50)**	**0.001**	**4.11**	**(1.50–11.38)**	**0.006**
Hypertension	**2.98**	**(1.17–7.59)**	**0.022**			
HIV	2.23	(0.60–8.38)	0.234			
Smoking	**3.77**	**(1.39–10.23)**	**0.009**	2.80	(0.98–8.03)	0.055
Male	2.11	(0.84–5.31)	0.111			
> 63 years	1.89	(0.73–4.90)	0.192			

*Note:* Values in bold indicate statistically significant results.

** Not included in final model as not significant (*p* ≥ 0.05).

**FIGURE 4:**
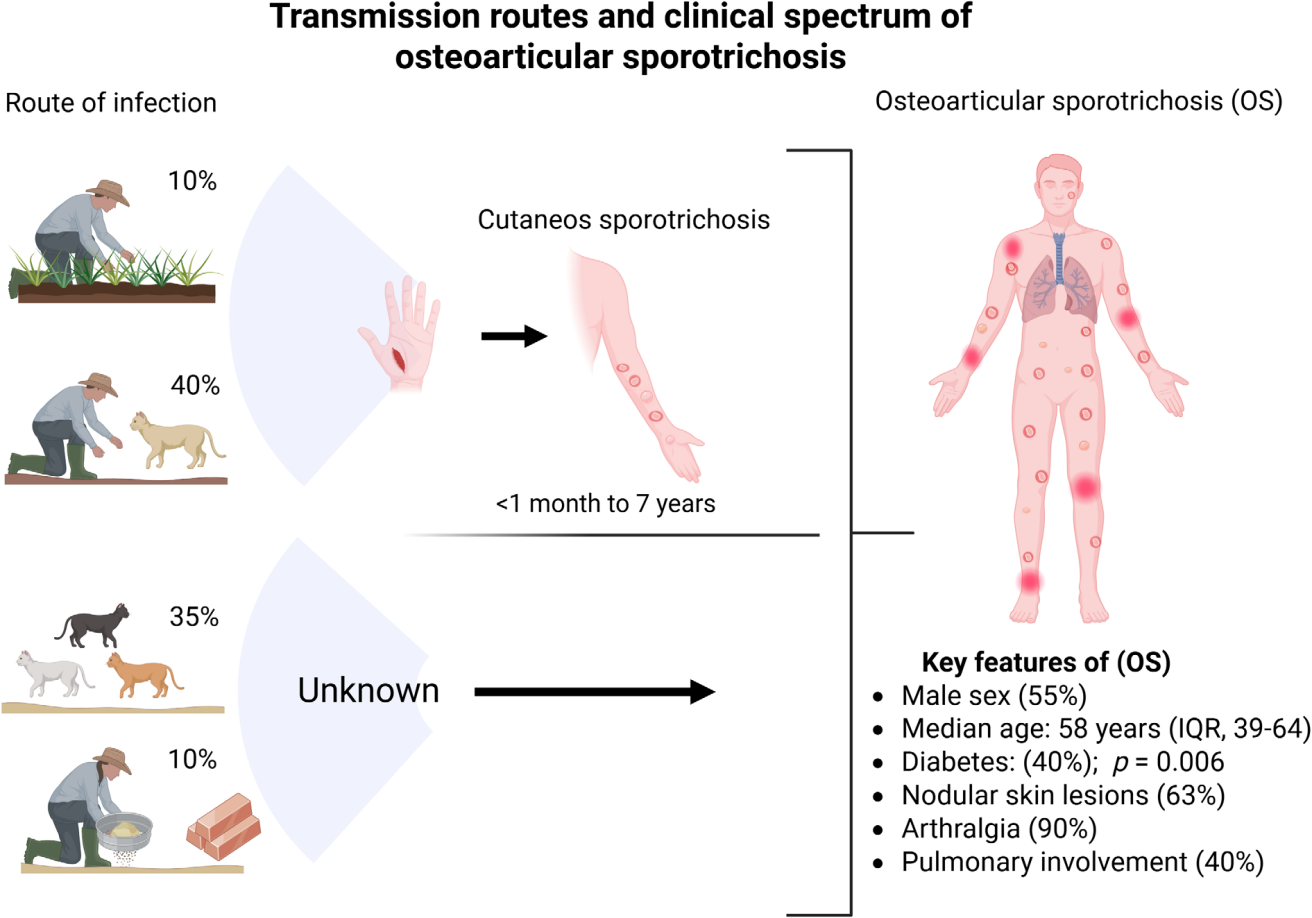
Summary of transmission routes of *Sporothrix* spp. and clinical characteristics of osteoarticular sporotrichosis. Created in BioRender. de Assis, D. (2026) https://BioRender.com/43jkuih.

Figure 4 summarizes the results from this study.

## 4 Discussion

The first case of OS in this study was documented in 2018, coinciding with the rise in feline and human sporotrichosis cases in the state of Minas Gerais, Brazil [5, 20, 21]. There was then a significant increase in OS cases, though this trend was interrupted between 2019 and 2021, when the hospital was exclusively dedicated to COVID‐19 patients. Since 2022, there has been a progressive rise in confirmed diagnoses, and the proportion of osteoarticular involvement in our cohort was high. The observed rate of bone involvement surpassed that reported in a large Brazilian OS series [11]. Overall, OS was primarily associated with high morbidity due to several factors, including late diagnosis, inadequate antifungal or surgical treatment, antifungal resistance, and immunosuppression. Mortality rates may have been affected by disseminated sporotrichosis and delays in diagnosis [22], particularly among PLWH [8, 23].

Notably, our data show that non‐traumatic contact with cats, whether or not involving direct handling, is associated with infection. This finding reinforces the role of transmission via contact with lesion secretions or respiratory droplets [24], as well as environmental sources, in *Sporothrix* infections [13, 14, 15, 25, 26]. Faeces from infected cats can contaminate the soil [27], where viable yeast cells may persist [25] and even revert to the saprophytic form under favourable conditions. Environmental inoculation may occur through skin lesions or via inhalation of conidia, which is considered the most likely route of transmission in pulmonary sporotrichosis [28, 29]. These findings highlight the importance of adopting a One Health approach to better elucidate the environmental reservoirs of *S. brasiliensis* and the potential for non‐zoonotic transmission [15], particularly among individuals with frequent soil exposure, such as gardeners and construction workers, as well as children who play outdoors.

The wide variation in the time between the onset of symptoms and diagnosis of OS reflects the diagnostic challenges of this clinical manifestation [13, 14, 30]. Osteoarticular mycoses are notoriously difficult to recognize, as diagnosis requires integrating clinical and epidemiological data with laboratory, imaging, and histopathological findings [11, 13, 30]. Moreover, the disease's slow progression contributes to further diagnostic delays [30].

As reported by Bayer et al., we have also observed a high positivity rate in fungal cultures of synovial fluid [13], highlighting the role of culture as a fundamental diagnostic tool in endemic areas, particularly in the absence of cutaneous lesions. A review study showed that skin lesion samples accounted for 11.7% of diagnoses among patients with arthritis, compared to 28.8% for synovial fluid samples. Among osteomyelitis cases, skin lesion samples accounted for 71.3% of diagnoses, compared to 4.6% from surgical specimens [12]. However, a confirmed diagnosis of osteomyelitis requires microbiological identification from a bone biopsy, ideally using at least three tissue samples to increase the likelihood of a positive result [10, 30].

The spectrum of osteoarticular involvement in sporotrichosis is broad and influenced by both host and pathogen‐intrinsic factors [30]. Osteomyelitis and arthritis can arise from exogenous sources, such as trauma; from contiguous extension of skin lesions, typically resulting in unifocal involvement affecting mainly the hands or feet; or from hematogenous dissemination to the long bones [12, 30]. The fungus may reach the joint space via the bloodstream and synovium, resulting in arthritis and osteolytic lesions, particularly in immunosuppressed patients [11, 31]. Additionally, tenosynovitis of the flexor or extensor tendons represents another form of contiguous involvement that can occur independently of bone or joint infection [11].

Patients with diabetes were 4 times more likely to develop OS than non‐diabetic individuals (*p* = 0.006), as previously reported [5, 12, 16, 32, 33, 34]. Diabetes may impair immune function by promoting chronic inflammation, disrupting cytokine signalling, and reducing phagocytic activity, thereby facilitating the dissemination of pathogens [35]. In addition to common complications such as vascular insufficiency, retinopathy, nephropathy, and neuropathy, diabetes leads to alterations in bone microarchitecture, including increased cortical porosity and reduced bone strength in patients with type 2 diabetes [36].

Therapeutic management is usually tailored according to the characteristics, extent, and severity of OS [17, 18, 37]. However, as reported in other studies [11, 16], the duration of itraconazole therapy and the cumulative doses of amphotericin B among cured patients often exceeded the recommended dose in current guidelines [17, 18, 37]. This finding suggests that standardized treatment recommendations may not fully account for the heterogeneity of osteoarticular sporotrichosis, particularly the differences between unifocal and multifocal forms resulting from disseminated infection, which may require more prolonged antifungal therapy. The standard itraconazole dosage is 400 mg/day, administered for 6 to 12 months or until complete clinical resolution is achieved. Amphotericin B is recommended as first‐line therapy for patients with extensive disease or those who fail to respond to itraconazole. In PLWH, antifungal therapy should be continued until the CD4^+^ T‐cell count exceeds 200 cells/μL [18, 37].

Determining the optimal time to discontinue treatment safely remains challenging, particularly in cases with significant osteoarticular destruction resulting from delayed diagnosis. Furthermore, more than half of the cases in our cohort required surgical intervention in addition to antifungal therapy. Previous studies have reported high cure rates with combined antifungal and surgical treatment [11, 12, 16]. Amputation has also been reported as a last resort for the treatment of osteomyelitis involving the fingers [11, 38]. Therefore, there is an urgent need for evidence‐based guidelines for the early diagnosis and comprehensive management of OS.

Antifungal susceptibility testing for itraconazole and amphotericin B (data not shown) indicated that the suboptimal therapeutic response was not due to in vitro resistance of *Sporothrix* isolates. Although some studies have reported comparable antifungal concentrations in synovial fluid and serum, evidence regarding the bone penetration of these agents remains limited [10]. Furthermore, Freitas et al. [39] showed that antifungal susceptibilities were similar among sequential isolates from patients with recalcitrant sporotrichosis. Therefore, other factors may contribute to failure, such as inadequate treatment adherence or suboptimal dosing and duration.

Sinovial fluid culture isolates of *S. brasiliensis* from a patient receiving itraconazole therapy exhibited yeast‐like cells, pseudohyphae, and filamentous forms simultaneously at 25°C and 37°C, suggesting morphological plasticity during infection. Evolutionarily conserved signalling pathways involved in fungal genetics, adaptive responses to distinct environmental conditions [40], and complex host–pathogen interactions [41] enable the fungus to sense and respond to environmental stimuli, often through coordinated changes in gene expression and morphogenetic transitions [42]. These regulatory changes may promote to fungal adhesion, cellular organization, and extracellular matrix production, ultimately facilitating the transition from planktonic cells to a structured biofilm community [42]. These mechanisms may also contribute to antifungal tolerance and persistence of infection. Similarly, Cognialli et al. reported patients presenting simultaneous yeast‐like and mycelial‐like structures in biopsy specimens, possibly influenced by local tissue conditions, including oxygen exposure and lower temperatures [43].

Biofilm formation is another critical survival mechanism that serves as a reservoir for persistent bone infections [10]. We hypothesize that *Sporothrix* spp. can form biofilms and adhere to the joint space by interacting with extracellular matrix components, such as type II collagen, fibronectin, and laminin [10, 42, 44]. Ex vivo studies demonstrate that fungal biofilms contribute to the pathogenesis of osteomyelitis by promoting both direct bone resorption and indirect immune‐mediated damage [44]. Additionally, fungal biofilms are more resistant to antifungal agents than planktonic cells [42].

The main limitation of this study is the clinical heterogeneity of this cohort. Patients were included at different stages of disease progression and with varying infection durations, which may have influenced the observed spectrum and severity of osteoarticular involvement. Furthermore, several patients were still undergoing antifungal therapy at the end of the study. Additionally, some patients were lost to follow‐up or died before confirmation of OS, particularly those with multisystem sporotrichosis, including pulmonary and neurological involvement.

In conclusion, OS is a debilitating infection often multifocal and associated with substantial morbidity, particularly in individuals with underlying comorbidities or immunosuppression. Delayed diagnosis leads to severe functional sequelae. Diabetes appears to be associated with increased risk. These findings underscore the urgent need for early recognition and prompt, targeted treatment to prevent irreversible damage in OS.

## Author Contributions

**Vanessa Caroline Randi Magalhães:** conceptualization, investigation, writing – original draft, methodology, visualization, writing – review and editing, validation, data curation, resources, formal analysis, software, project administration. **Alexandre Sampaio Moura:** writing – review and editing, investigation, validation, supervision, resources, data curation. **Renata Eliane de Ávila:** investigation, validation, writing – review and editing, resources. **Amanda Cristina Silva Tardelli Pizarro:** investigation, writing – review and editing, resources. **Jorge Miguel Schettino César:** investigation, writing – review and editing, resources. **Ana Cláudia Lyon:** investigation, writing – review and editing, resources. **Silvia Hees Carvalho:** investigation, writing – review and editing, resources. **Diana Manzi Viegas:** investigation, writing – review and editing, resources. **Virginia Antunes Andrade:** investigation, writing – review and editing, resources. **Nalu Teixeira Aguiar Peres:** writing – review and editing, validation, resources, conceptualization. **Fábio do Nascimento Sales:** writing – original draft, writing – review and editing, investigation. **Maria Isabel Azevedo:** writing – review and editing, validation, resources, supervision, data curation, methodology. **Salene Angelini Colombo:** writing – review and editing, investigation, validation, data curation, methodology. **Daniel Assis Santos:** resources, project administration, writing – review and editing, validation, conceptualization, funding acquisition, methodology, supervision. **Maria Rita Teixeira Dutra:** investigation, writing – review and editing, resources.

## Funding

This study was supported by Fundação de Amparo a Pesquisa do Estado de Minas Gerais‐FAPEMIG (apq‐05368‐26), Coordenação de Aperfeiçoamento de Pessoal de Nível Superior‐CAPES, Conselho Nacional de Desenvolvimento Científico e Tecnológico‐CNPq/MS (402200/2021‐7; 408540/2022‐2), Brazilian Ministry of Health (440010/2018‐7). V.C.R.M. (BPG‐00113‐23) FAPEMIG. D.A.S. (303762/2020‐9) is a research fellow of the CNPq.

## Conflicts of Interest

The authors declare no conflicts of interest.

## Data Availability

The data that support the findings of this study are available from the corresponding author upon reasonable request.
